# NLOS Correction/Exclusion for GNSS Measurement Using RAIM and City Building Models

**DOI:** 10.3390/s150717329

**Published:** 2015-07-17

**Authors:** Li-Ta Hsu, Yanlei Gu, Shunsuke Kamijo

**Affiliations:** Institute of Industrial Science, The University of Tokyo, 4-6-1 Komaba, Meguro-ku, Tokyo 153-8505, Japan; E-Mails: guyanlei@kmj.iis.u-tokyo.ac.jp (Y.G.); kamijo@iis.u-tokyo.ac.jp (S.K.)

**Keywords:** GNSS, GPS, NLOS, multipath, RAIM, 3D maps, building models, urban canyon, consistency check, particle filter

## Abstract

Currently, global navigation satellite system (GNSS) receivers can provide accurate and reliable positioning service in open-field areas. However, their performance in the downtown areas of cities is still affected by the multipath and none-line-of-sight (NLOS) receptions. This paper proposes a new positioning method using 3D building models and the receiver autonomous integrity monitoring (RAIM) satellite selection method to achieve satisfactory positioning performance in urban area. The 3D building model uses a ray-tracing technique to simulate the line-of-sight (LOS) and NLOS signal travel distance, which is well-known as pseudorange, between the satellite and receiver. The proposed RAIM fault detection and exclusion (FDE) is able to compare the similarity between the raw pseudorange measurement and the simulated pseudorange. The measurement of the satellite will be excluded if the simulated and raw pseudoranges are inconsistent. Because of the assumption of the single reflection in the ray-tracing technique, an inconsistent case indicates it is a double or multiple reflected NLOS signal. According to the experimental results, the RAIM satellite selection technique can reduce by about 8.4% and 36.2% the positioning solutions with large errors (solutions estimated on the wrong side of the road) for the 3D building model method in the middle and deep urban canyon environment, respectively.

## 1. Introduction

Urban canyon is one of the most challenging environments for global navigation satellite system (GNSS) positioning. The high buildings and skyscrapers can easily block or reflect the GNSS signal to induce the signal delay, which are well-known as the multipath effect and non-line-of-sight (NLOS) receptions. These signal reflection effects limit the application of the GPS positioning in city urban area, for example, the pedestrian localization for the applications of the pedestrian’s safety in the intelligent transportation system (ITS). The technology of autonomous driving also requires accurate and reliable positioning services in urban areas. Many studies are therefore focused on improving GNSS positioning performance in the degraded environments. Conventionally, the multipath can be mitigated using sophisticated antenna designs [1,2] and receiver-based discriminator designs [3,4]. The multipath mitigation methods mentioned above have little improvement on NLOS reception. Approaches to NLOS mitigation are therefore needed. With the aid of receiver dynamic provided by inertial sensor, tightly and ultra-tightly coupled GPS/INS integrations are proposed to mitigate the multipath and NLOS effects [5,6,7,8,9]. A multipath estimation, which is based on integration between GNSS receiver and LiDAR sensor, is proposed [10]. Novel receiver-based techniques are also proposed to detect multipath and NLOS signals [11,12].

**Figure 1:**
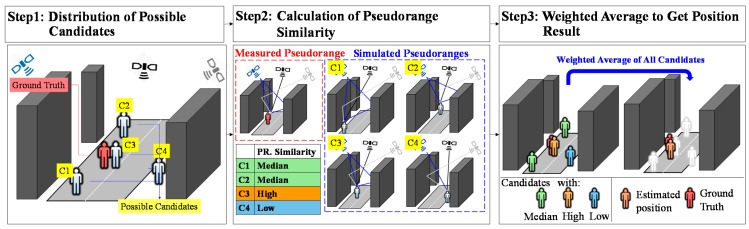
The idea of the developed 3D building model based positioning method.

One of the newly proposed solutions is to take advantage of the city building models [13,14,15,16,17,18,19,20,21,22] to mitigate, detect or even correct the reflection signals. In 2013, The research team of The University of Tokyo developed a particle filter based positioning method using a basic three-dimension (3D) city building to estimate the positioning result of commercial GNSS single frequency receiver [23,24,25]. Figure 1 shows the basic idea of the proposed 3D building model positioning method.

The proposed method first distributes the random position candidates, and then calculates the pseudorange similarities between raw pseudorange measurements and the simulated pseudoranges (estimated by the help of the 3D building models and ray-tracing technique [26]) for each candidate. The likelihood of each candidate is based on its pseudorange similarity. Finally, the weighted average of the positions of the candidates is regarded as the estimated position result. The studies that corrected NLOS and used it as an additional measurement, including the proposed building model based positioning method, assumes the travelling path of the NLOS is single reflected [18,20,25]. In the case of general urban canyons, this assumption should be correct in most cases. Because of the dense and modern buildings in middle or deep urban canyons, the possible reflection paths of GNSS signals are increased dramatically. As a result, a double or multiple reflected NLOS signal can be easily observed. These multiple reflection signals increase the difficulty and immense computational load of using the signal ray racing to track the signal reflecting routes. As a result, abnormal signal exclusion is required in the positioning method. A practical NLOS signal exclusion algorithm, called consistency check, has been proposed recently [27]. This consistency check follows the idea of receiver autonomous integrity monitoring (RAIM) fault detection and exclusion (FDE) to exclude abnormal signals by the pseudorange residual [28,29]. This paper is inspired by the consistency check used in the weighted least square (WLS) method. Instead of applying the RAIM in the conventional positioning method, this paper applies the RAIM in the developed 3D building model based positioning method to exclude the abnormal reflection signals.

Accordingly, this paper is organized as follows: The related works on GNSS 3D map method and RAIM FDE are given in Section 2. A brief introduction of the 3D building model positioning method is introduced in Section 3. Detail of the developed RAIM FDE is presented in Section 4. The experimental setup and results are shown in Section 5. Finally, the conclusions and future work of this paper are summarized in Section 6.

## 2. Related Works

Recently, using 3D building model as aiding information to mitigate or exclude the multipath and NLOS effects has become a popular topic of study. The metric of NLOS signal exclusion using an elevation-enhanced map, extracted from a 3D map, is developed and tested using real vehicular data [21]. An extended idea of identifying NLOS signals using infrared camera set at an automotive vehicle was suggested [22]. The potential of using a dynamic 3D map to design a multipath exclusion filter for a vehicle-based tightly-coupled GPS/INS integration system was studied in [14]. A forecast satellite visibility based on a 3D urban model to exclude NLOS signals in urban areas was developed in [15]. The above approaches aim to exclude the NLOS signal; however, the exclusion is very likely to cause a horizontal dilution of precision (HDOP) distortion scenario, due to the blockage of buildings along the two sides of streets. In other words, the lateral positioning error would be much larger than that of the along track direction. As a result, approaches that applied multipath and NLOS signals as measurements become essential. The shadow matching method uses 3D building models to predict the satellite visibility and to compare it with measured satellite visibility to improve the cross street positioning accuracy [19]. NLOS delay estimation by a 3D map-based particle filter, which is used in this paper, was proposed and tested in with dynamic pedestrian experiments [23,24]. The benefit of the quasi-zenith satellite system (QZSS) L1-submeter-class augmentation with integrity function (L1-SAIF) and the rising of the multi-GNSS to the 3D map-based positioning method are evaluated [25,30], respectively.

RAIM is a GPS receiver self-checking algorithm for the fault of navigation solution based on redundant measurements and proposed by [28]. RAIM is well-known for its capability of detecting the error caused by mis-modeling. As a result, researchers from Stanford University proposed applying weighted RAIM to capture mis-modeling of ionospheric and satellite orbit/clock corrections [31]. A detailed algorithm and simulation of RAIM availability is released by Imperial College University [32]. Recently, the intention of using RAIM to deal with multipath and NLOS effect is increased. A scenario of only five GPS satellites in view is studied to increase the availability of RAIM [33]. A consistency checking algorithm using RAIM concept is developed to mitigate the multipath effects [34]. Toyota ITS team also releases a report to show the effeteness of RAIM to exclude multipath range error for vehicle application [35]. Discussions of using multi-GNSSs RAIM, including GPS/GLONASS/Beidou/QZSS, are described in [36]. In 2015, a simulation result of multiple fault exclusion with large number of pseudorange measurements is released, showing its capability in the area with high probability of measurement fault [37]. This multiple fault exclusion based on L1 norm minimization [38] is promising to exclude strong multipath effects and NLOS reception. It is very interesting to evaluate this multiple fault exclusion algorithm in the previous proposed 3D map pedestrian positioning method. This RAIM multiple fault detection algorithm is expected to exclude the abnormal NLOS signal. Thus, it is the objective of this paper.

## 3. 3D Building Model Based Positioning Method

**Figure 2:**
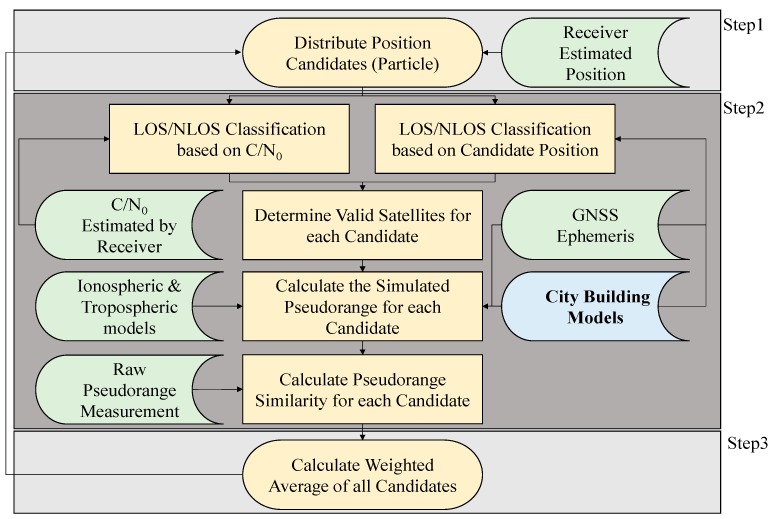
Flowchart of the developed 3D building model based positioning method.

The flowchart of the proposed 3D map-based pedestrian positioning method is shown in Figure 2.

As shown in Figure 2, this method firstly implements a particle filter to distribute position candidates (particles) around the receiver estimated position, which is assumed to be within about 15 m of the ground truth. When a candidate position is given, the proposed method can evaluate whether each satellite is in LOS, multipath or NLOS, by applying the ray-tracing procedure with a 3D building model. According to the signal strength, namely carrier to noise ratio (C/N_0_), the satellite could be roughly classified into LOS, NLOS and multipath scenarios. The signal classification used in the proposed method is shown in Table 1 [25]. The thresholds of the signal strength are set based on the empirical experience [25]. If the signal classifications are inconsistent between each other, the satellite will not be used for the candidate. If they are consistent, the simulated pseudorange of the satellite for the candidate will be calculated.

**Table 1:** Rule of signal classification for a candidate based on signal strength and ray-tracing [25].

Signal Strength (C/N_0_)	Ray-Tracing	Valid Satellite Type
LOS (>40 dB-Hz)	LOS	LOS
NLOS (<30 dB-Hz)	NLOS	NLOS
LOS (>40 dB-Hz)	NLOS	Invalid
NLOS (<30 dB-Hz)	LOS	Invalid
Unknown (30 dB-Hz < C/N_0_ < 40 dB-Hz)	LOS	Multipath (LOS if no reflection path is found)
Unknown (30 dB-Hz < C/N_0_ < 40 dB-Hz)	NLOS	NLOS

**Figure 3:**
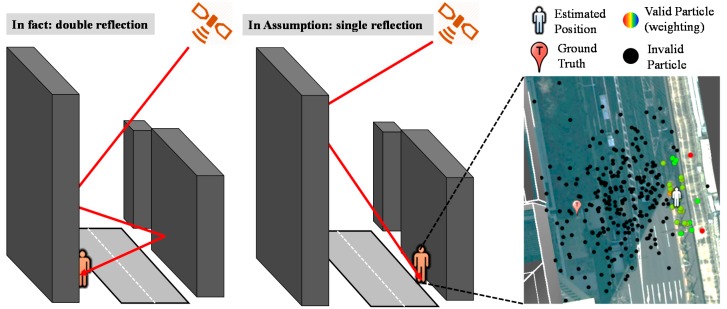
Illustration of the devastated effect caused by double reflected non-line-of-sight (NLOS).

In the LOS case, simulated pseudoranges can be estimated as the distance of the direct path between the satellite and the assumed position. In the multipath and NLOS cases, the simulated pseudoranges can be estimated as the distance of the reflected path between the satellite and the candidate position via the building surface. Ideally, if the position of a candidate is located at the true position, the difference between the simulated and measured pseudoranges should be zero. In other words, the simulated and measured pseudoranges should be identical. Therefore, the likelihood of each valid candidate is evaluated based on the pseudorange difference between the pseudorange measurement and the simulated pseudorange simulated by 3D building models and ray tracing. Finally, the expectation of all the candidates is the estimated positioning of the proposed map method. The proposed method is able to find the optimum position through a dedicated optimization algorithm of the above assumptions and evaluations. The detail algorithm of the particle filter using 3D city building models can be found in [23,24,25]. However, this particle filter still suffers from the effect of multiple reflected NLOS in the process of generating simulated pseudorange. In the developed method, a double reflected NLOS signal is devastating because of the assumption of single reflection. In a deep urban canyon environment, namely high buildings located on both sides of a street, the single reflection assumption is not always correct. If a double reflected NLOS is received, the developed method will estimate the user location at wrong side of street due to single reflection limit as shown in Figure 3. The candidates become invalid in the correct side of the street due to the incorrect assumption. As a result, the developed method will estimate a fault positioning solution.

## 4. Abnormal Measurement Exclusion Using RAIM Multiple Fault Detection and Exclusion

**Figure 4:**
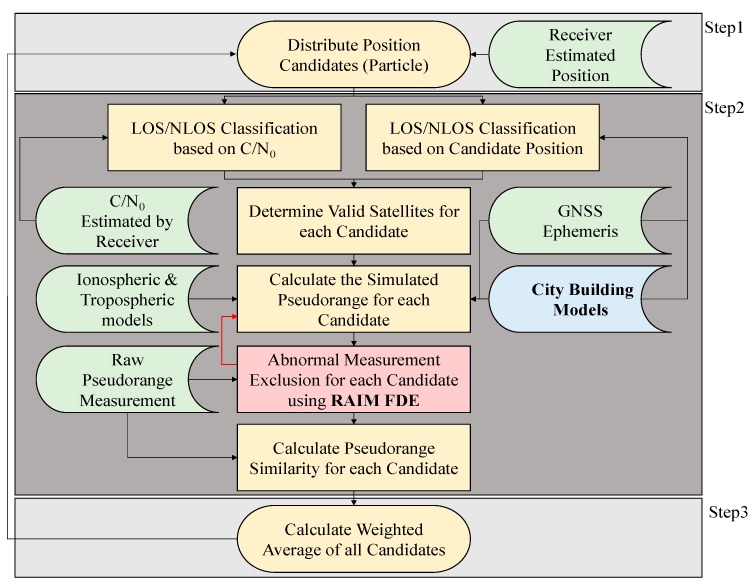
Flowchart of the particle filter using 3D city building models and receiver autonomous integrity monitoring (RAIM) multiple fault detection and exclusion (FDE).

**Figure 5:**
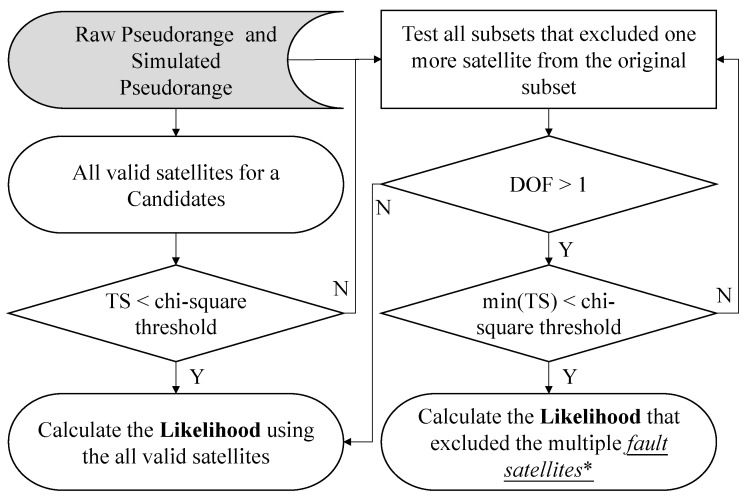
RAIM multiple fault exclusion used in the proposed 3D map method. TS, test statistic; and DOF, degree of freedom.

The idea of RAIM FDE is to exclude the satellite with larger pseudorange residual. As mentioned earlier, it is capable of excluding abnormal signals. An illustration of the devastated effect of the particle filter is depicted in Figure 3. This paper therefore proposes the idea of RAIM to overcome the drawback. Figure 4 shows the flowchart of the use of the proposed RAIM FDE in the 3D map method. The red-frame of Figure 4 is the newly proposed method in this paper. As shown in Figure 4, the RAIM FDE will be used after calculating the simulated pseudorange of a candidate. It is interesting to note the idea of the pseudorange difference of each candidate in the proposed 3D map positioning method is similar to the pseudorange residual of the RAIM algorithm. In the proposed method, the pseudorange difference is defined as the pseudorange measurement minus the simulated pseudorange. The simulated pseudorange is calculated as:

(1) $${\hat{\rho }}_{n}^{\left(i\right)}=R_{n}^{\left(i\right)}+c\left(\text{δ}t^{\text{r}\left(i\right)}-\text{δ}t_{n}^{\text{sv}}\right)+I_{n}+T_{n}+{\text{ε}}_{n}^{\text{refl}\left(i\right)}$$

where *n* denotes the index of the satellite, *i* denotes the index of a position candidate (particle), $R_{n}^{\left(i\right)}$ denotes geometric distance between the satellite *n* and the candidate *i*, *c* denotes the speed of light, the satellite clock and orbit offset $\text{δ}t_{n}^{sn}$ are corrected using the satellite broadcast model. The ionospheric delay *I* and the tropospheric delay *T* are obtained from the Klobuchar and Saastamoinen models, respectively. The reflection delay is estimated by the ray tracing and city building models [23,24,25]. The receiver clock offset, $\text{δ}t^{r\left(i\right)}$, for the candidate is modified to minimize the difference between the simulated set and the measured set. If an abnormal signal is used in the calculation of the receiver clock bias minimization, the optimized receiver clock bias will be inaccurate. The flowchart of the RAIM multiple fault exclusion in the proposed method is shown in Figure 5.

Firstly, all valid satellites are selected into a subset. The valid satellite means a satellite that is classified to same signal type by both the C/N_0_ and ray-tracing classifications. Afterwards, the pseudorange difference (${\text{ε}}_{n}^{pr}$) is calculated as:

(2) $${\text{ε}}_{n}^{pr}={\text{ρ}}_{n}-{\hat{\rho }}_{n}^{\left(i\right)}\left(\partial t_{optimized}^{\left(i\right)}\right)$$

where $\partial t_{optimized}^{\left(i\right)}$ is the receiver clock bias for a candidate based on the previous developed optimization method [23]. The root of the sum of the squared pseudorange difference, which defined as a test statistic (TS) in this paper, is as:

(3) $$TS=\sqrt{\sum_{n=1}^{N^{sim}}{\left({\text{ε}}_{n}^{pr}\right)}^{2}}$$

where $N^{sim}$ denotes the number of simulated pseudoranges. This paper assumes the pseudorange difference is normally distributed zero mean random variable. Thus the pseudorange difference can be teste by the chi-square test according to appropriate degree of freedom (DOF) and probability of false alarm. The DOF is calculated using Equation (4) because it requires at least one satellite from the same GNSS constellation to calculate the optimized receiver clock bias.

(4) $$DOF=N^{sim}-1$$

Theoretically speaking, two satellites from the same constellation mean the DOF equals 1, which can be checked by the consistency of receiver clock bias. However, this check could be difficult in the practical implementation. For example, in the case of two satellites are received, one is a clean LOS signal and the other one is abnormal NLOS signal. In this case, the DOF is 1 theoretically, and it should be checked by the constraint of receiver clocks. However, it is very difficult for the algorithm to identify which one is clean or abnormal. Note that the optimal receiver clock bias calculated by the proposed method is also estimated by the two satellites. In this case, it has a high probability that the consistency check excludes a clean LOS signal. Thus, the proposed method uses the RAIM when at least three satellites from a same constellation are received. In the case of two LOS signals and one abnormal NLOS signal are received, the proposed method is capable of excluding the abnormal NLOS. Even if in a special case that one LOS and two abnormal NLOS are received, the two abnormal NLOS do not seem to agree with each other. Generally speaking, the LOS will not be excluded by the proposed RAIM algorithm. Thus, the proposed RAIM is only used when the DOF is larger than 1. The probability of false alarm (P_FA_) used in this paper is 10^−4^. By giving the P_FA_, the value of the chi-square threshold (*T*) can be calculated as [28,31,39]:

(5) $$1-P_{FA}=\frac{1}{\text{Γ}\left(DOF/2\right)}\int_{0}^{T^{2}}e^{-s}s^{\frac{DOF}{2}}ds$$

To reduce the computational load, the value of the chi-square (consistency) threshold is calculated in advance and saved in the program as listed in Table 2.

**Table 2:** Values of chi-square threshold (probabilities of false alarm is 10^−4^) for given degree of freedom (DOF).

DOF	1	2	3	4	5	6	7
**Value**	22.93	27.64	30.67	33.37	35.89	38.25	40.53

If the test statistic is smaller than the value of the chi-square threshold, all the valid satellites will be used in the position estimation. On the other hand, if the test statistic is larger than the chi-square threshold, it implies one of the valid satellites used in the subset may be deteriorated by double or multiple reflected NLOS. In this case, the satellite exclusion by RAIM is required. We apply the greedy search algorithm to remove the satellite one by one until it meets the chi-square threshold [37]. The fault exclusion used in this paper assumes only one satellite is biased at one iteration. It is achieved by trying every subset.

(6) $$TS\left(k\right)=\sqrt{\sum_{n=1,\;n\neq k}^{N^{sim}}{\left({\text{ε}}_{n}^{pr}\right)}^{2}}$$

(7) $$k_{\mathrm{excluded}}=\arg\min_{k=1\cdot \cdot \cdot N^{\mathrm{sim}}}TS(k)$$

Again, if the *TS(K)* is smaller than the chi-square threshold, the *k*th satellite will be excluded if the test statistic of the subset without it is the minimum. As a result, the developed method can exclude the abnormal NLOS signal to estimate a more accurate position solution. However, if the *TS(K)* is still larger than the threshold, we exclude the satellite measurement that has the largest impact on the test statistic. In other words, the $k_{\mathrm{excluded}}$ satellite will be excluded from the subset of the next iteration. The iteration will stop until the remaining set of the measurements is self-consistent or the DOF is not enough.

## 5. Experimental Results and Discussion

**Figure 6:**
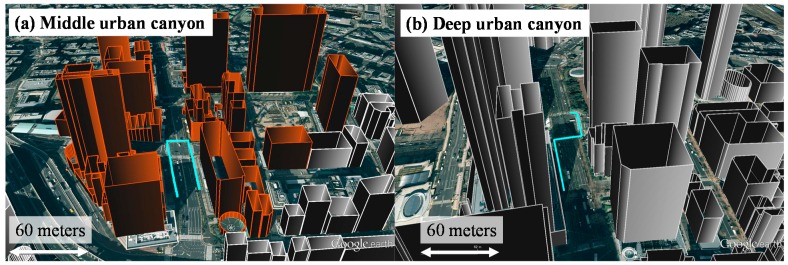
Constructed 3D building models in the middle and deep urban canyons. The cyan lines indicate the pedestrian walking trajectories in the dynamic experiments.

This paper selects the Hitotsubashi and Shinjuku area in Tokyo to be the experimental middle and deep urban canyons, respectively. The constructed 3D building models are shown in Figure 6. The single point GPS positioning results, such as weighted least square (WLS), are poor in the two areas. The tests in this paper are performed at two sides of a street and a road intersection. The cut-off angle is 20° in this paper. The data were collected on 5 November 2014, 14 December 2014 and 26 January 2015. The durations of both data are about 210 s. This paper uses a commercial grade receiver, u-blox EVK-M8 GNSS model. The u-blox receiver is set to output pseudorange measurements and positioning result every second. GPS, GLObal NAvigation Satellite System (GLONASS) and QZSS measurements are used in this paper. The quasi ground truth is generated using a topographical method. The video cameras are set in the 18th and 9th floors of a building near the Hitotsubashi and Shinjuku area, respectively, to record the travelled path. The video data output by the cameras are used in combination with one high-resolution aerial photo we bought to get the ground truth data. The aerial photo is 25 cm/pixel and therefore the error distance for each estimate can be calculated. This paper evaluated the lateral positioning error. There are two conventional positioning methods that used for the purpose of comparison. The first one is WLS, and the weighting matrix follows the manual of RTKLIB, which is an open source program [40]. The second one is the WLS using only the LOS visible satellites. The LOS satellites are determined by the ray-tracing results based on the ground truth trajectory. The performance metrics used are the mean and standard deviation of the lateral error and the availability. Availability means the percentage of solutions in a fix period. For example, if a method outputs 80 epochs in a 100 s period, the availability of the method is 80%. Note that the positioning solution will be excluded if its error is larger than 100 m. In addition, the satellite will be excluded if its C/N_0_ is less than 25 dB-Hz.

### 5.1. Middle Urban Canyon

**Figure 7:**
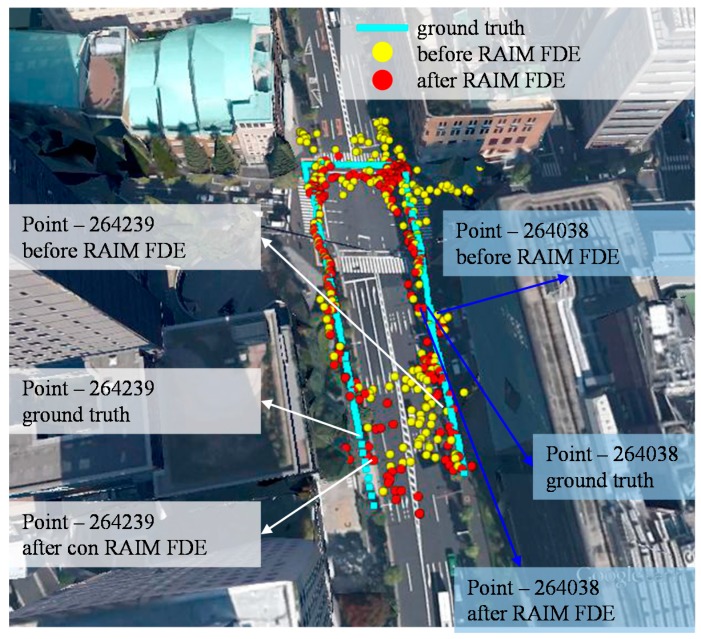
Results of the proposed 3D map positioning method before and after using the RAIM satellite selection method in a middle urban canyon.

Figure 7 shows the dynamic pedestrian positioning results of the proposed 3D map in the middle canyon.

The yellow and red dots indicate the position solutions of the 3D map method before and after using the RAIM satellite selection method, respectively. As can be seen in Figure 7, it is difficult to find which side of the street the pedestrian is walking on by the yellow dots at many different points. On the contrary, the red ball, the result of applying RAIM, is much closer to each side of the street. In the intersection, the reflection path is more complicated than that in the link. The 3D map method could sometimes estimate the incorrect NLOS reflection path, which resulted in inaccurate positioning result. The proposed RAIM method can also reduce this defect because the satellite that inconsistent between pseudorange measurement and simulated pseudorange will be excluded. The statistic of Figure 7 is listed in Table 3. Comparing the two, the positioning error is about 3.97 and 2.96 m before and after using the RAIM, respectively. The improvement by RAIM not only can be found in terms of mean but also standard deviation. This improvement indicates the proposed RAIM makes the 3D map method more accurate and precise. The conventional positioning methods cannot achieve a similar performance even if it only uses the LOS satellite measurements.

**Table 3:** Lateral positioning performance of the conventional method and the proposed 3D map method before and after using the RAIM method in the middle urban canyon.

Methods	Mean (m)	Std (m)	Availability
Conventional positioning method (WLS)	24.28	28.61	97.23%
WLS using only LOS	12.57	13.38	53.36%
3D map method	3.97	3.97	100%
3D map method with RAIM FDE	2.96	2.44	100%

**Figure 8:**
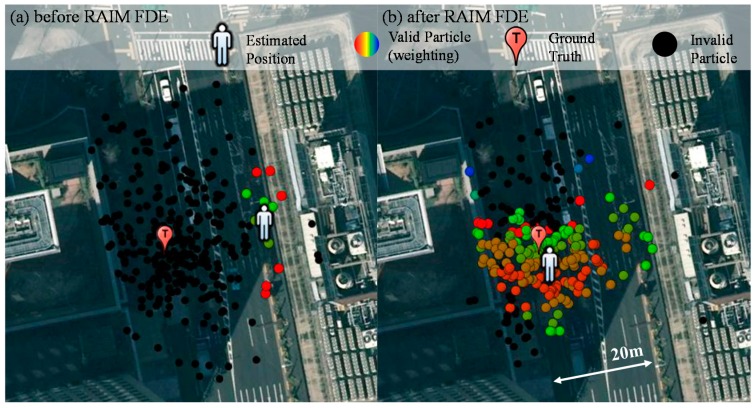
Weighting of all the particles of the point 264,239 before (**a**) and after RAIM (**b**). The color of the particle indicates the weighting of each particle. Red and blue indicates the highest and lowest weighting, respectively.

**Figure 9:**
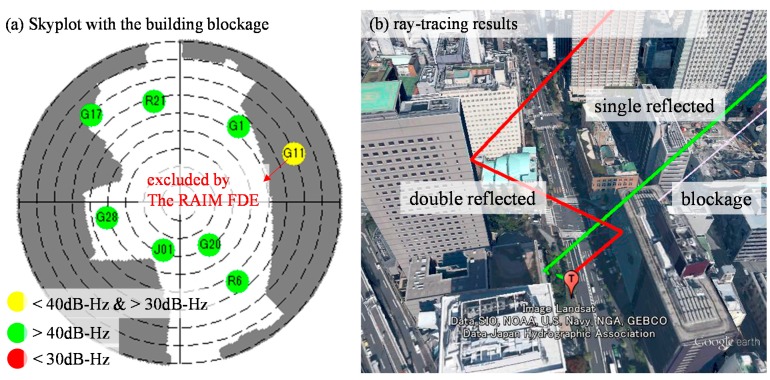
(**a**) Skyplot of point 264,239, and the dark grey color indicates the view blocked by surrounding buildings; (**b**) Ray-tracing result of point 264,239 based on the ground truth position.

To demonstrate the performance improved by the RAIM, we select two typical points in this dynamic data, point 264,038 and 264,239. To observe the left side of Figure 7 (point 264,239), the positioning result before and after applying RAIM is very different. The red ball is much closer to the ground truth than yellow ball. The position solution of the 3D map method is calculated by:

(8) $$x=\frac{\sum_{i}{\text{α}}^{\left(i\right)}P^{\left(i\right)}}{\sum_{i}{\text{α}}^{\left(i\right)}}$$

where *x* denotes the position estimated, α denotes the weighting of a particle and *P* denotes the position of a particle. Hence, the weighting of the particles is very essential for the proposed method. Figure 8 shows the weighting of the particle of point 264,239 before and after applying the RAIM. It is obvious that the weighting of the particle behaviors are very different in Figure 8a,b. All of the particles in the left side of Figure 8a are invalid; on the contrary, most of particles in the left side of Figure 8b are valid. Figure 9a,b shows the skyplot and ray-tracing result of point 264,239, respectively. Before applying RAIM technique, the GPS PRN 11 is used in all the particles in the left side. Its simulated pseudorange is assumed to be the single reflection (green line in Figure 9b). However, its pseudorange measurement indicates it is a double reflected NLOS (red line in Figure 9b).

**Figure 10:**
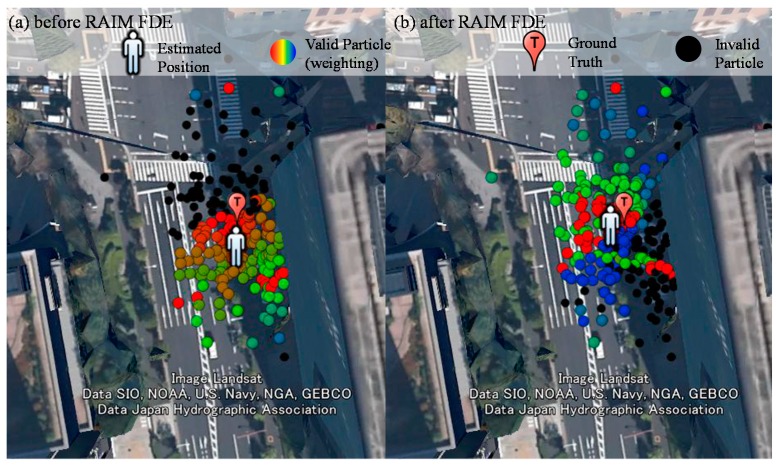
Weighting of all the particles of point 264,308 before (**a**) and after RAIM (**b**).

**Figure 11:**
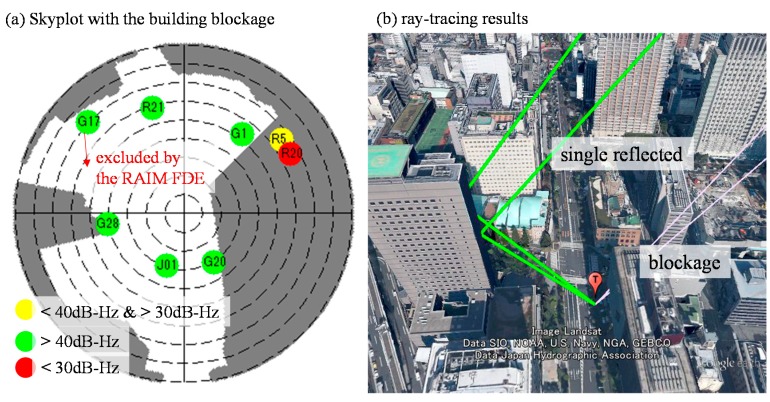
(**a**) Skyplot of point 264,308, and the dark grey color indicates the view blocked by surrounding buildings; (**b**) Ray-tracing result of point 264,308 based on the ground truth position.

As a result, the pseudorange difference between the simulated and raw pseudorange is large. This large difference results in the particle on the left side of the street in Figure 8a to become invalid. With the aid of the proposed RAIM, GPS 11 is excluded from the particles of Figure 8b. This exclusion facilitates the proposed 3D map method to estimate the correct side of the street in this case. With regard to point 264,038, the positioning results before and after applying RAIM are similar, as shown in the right side of Figure 7. Figure 10 shows the weighting of all the particles in this point. The GLONASS satellites 5 and 20 are both single reflected NLOS, as indicated by the pseudorange measurements, which are similar to their simulated pseudorange, as shown in Figure 11b. In this case, both the GLONASS NLOS satellites’ measurements are safe to use. Thus, the position estimated by the 3D map method standalone is close to the ground truth.

After applying the RAIM, GPS 17 is excluded from the upper particles in Figure 10b. GPS PRN 17 is a relatively low elevation satellite, which usually contains stronger multipath effect. Therefore, these particles become valid. The estimated position of the proposed 3D map method with the RAIM is slightly closer to the ground truth compared to that of the 3D map method standalone. Hence, the proposed RAIM algorithm can, not only exclude the double reflected NLOS, but also strong multipath signals.

### 5.2. Deep Urban Canyon

**Figure 12:**
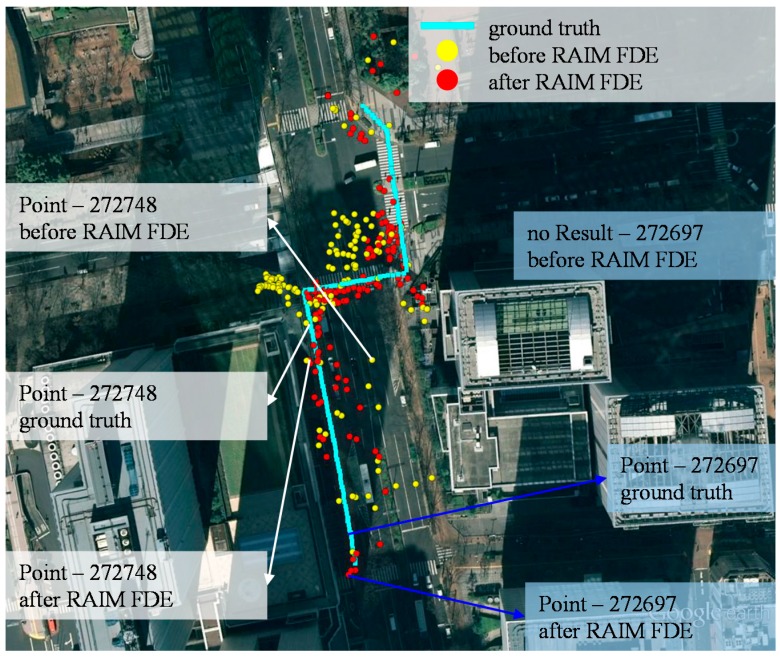
Results of the proposed 3D map positioning method before and after using the RAIM satellite selection method in the deep urban canyon.

**Figure 13:**
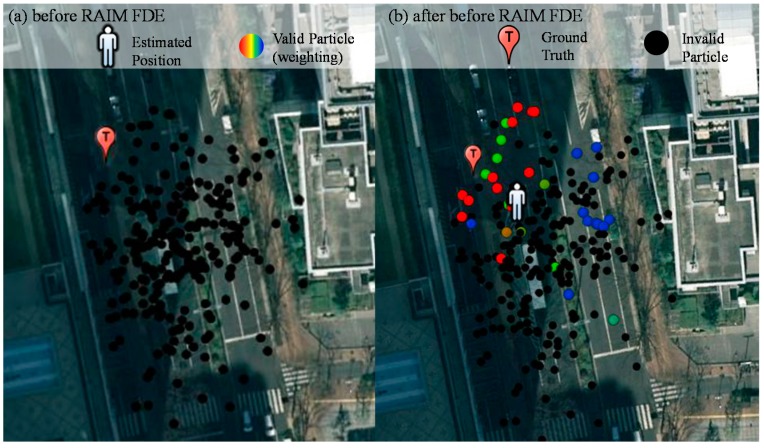
Weighting of all the particles of point 272,697 before (**a**) and after RAIM (**b**).

Figure 12 shows the dynamic pedestrian positioning results of the proposed 3D map method in the deep canyon. As can be seen in Figure 12, it is difficult to understand the trajectory of the pedestrian using the 3D map method (yellow dots). The 3D map method even gives a result with the wrong side of the street at many points. After applying the RAIM FDE, the positioning results of the 3D map method became much closer to the ground truth. Table 4 lists the lateral positioning performance of the conventional and 3D map methods. Both the conventional methods cannot provide accurate positioning service. It is interesting to note the WLS using only LOS has low availability because of the insufficient number of LOS satellites. Comparing the 3D map method before and after applying the RAIM FDE, there are about 4.9 and 2.1 m of improvements in terms of positioning mean error and standard deviation, respectively. Figure 12 shows that the positioning results before applying RAIM have about 12 m of lateral positioning error at point 272,748. After applying the RAIM, the positioning error is reduced to about 2 m. The improvements are due to the exclusion of the double reflected NLOS and strong multipath effects, as previous demonstrated in Figure 8 and Figure 10. The availability is also increased after applying the RAIM. Point 272,697 in Figure 12 is an example of the increase of the availability. The particle weighting distribution of the point 272,697 is shown in Figure 13.

**Table 4:** Lateral positioning performance of the conventional method and the proposed 3D map method before and after using the RAIM method in the deep urban canyon.

Methods	Mean (m)	Std (m)	Availability
Conventional positioning method (WLS)	23.99	20.10	55.02%
WLS using only LOS	13.21	20.32	10.58%
3D map method	8.78	5.62	73.68%
3D map method with RAIM FDE	3.85	3.56	83.16%

As can be seen in Figure 13a, there are no valid particles before applying the RAIM. The reason is the 3D map method cannot find a single reflected NLOS transmission length that is similar to that of the multiple reflected NLOS. If the NLOS satellite is excluded, the weighting of the particle is shown in Figure 13b. As a result, the positioning result becomes close to the ground truth. In Table 3 and Table 4, we can see that the proposed RAIM abnormal satellite exclusion method can reduce lateral positioning error greatly in both middle and deep urban canyons.

### 5.3. Histogram Study of the Lateral Positioning Error of the Proposed 3D Building Model Based Positioning Method

**Figure 14:**
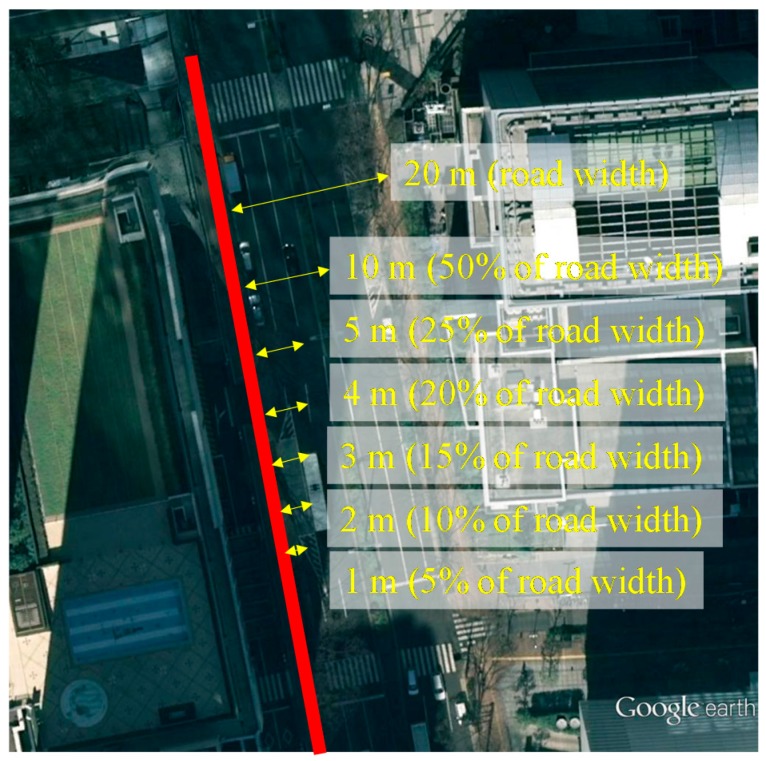
Demonstration of the bins of the histogram of the lateral positioning error. The red line indicates the pedestrian walking trajectory.

**Figure 15:**
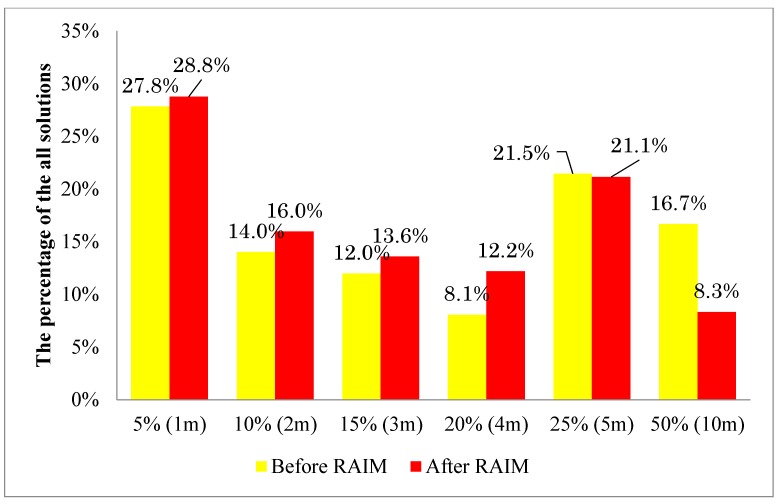
Histogram of the lateral positioning error of the proposed 3D map method before and after using the RAIM satellite selection method in the middle urban canyon.

**Figure 16:**
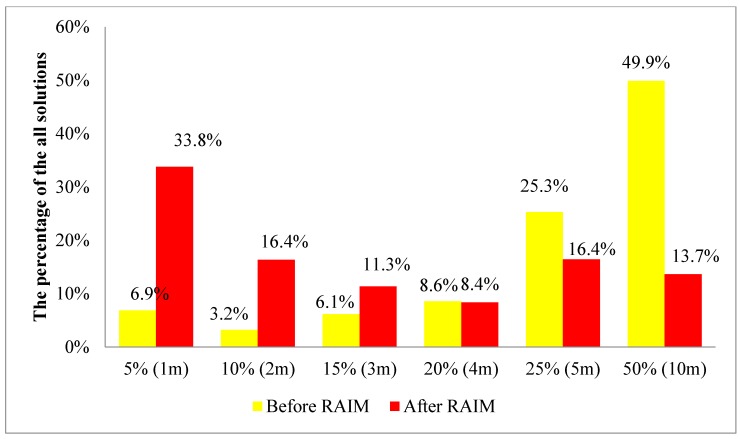
Histogram of the lateral positioning error of the proposed 3D map method before and after using the RAIM satellite selection method in the deep urban canyon.

This subsection focuses on the statistical comparison of the proposed 3D map method before and after using the RAIM satellite selection technique. The GNSS positioning solution is large in lateral direction, especially in urban canyon environments. It is essential to study the histogram of the lateral positioning error. This paper defines the bins of the histogram as the percentage of the road width, as shown in Figure 14. The experiments are conducted in both middle and deep urban canyon. The road widths of the experimental place are about 20 m. As can be seen, if the lateral positioning error is larger than 50% of the road width, namely larger than 10 m, the pedestrian is estimated on the wrong side of the road. The point with positioning error less than 3 m are classified as accurate solutions. In this experiment, the pedestrian walks the street back and forth. The trajectory is as the red line in Figure 14. The lengths of the data used are about 20 min, which contains about 1200 epochs. The results in the middle and deep urban canyon are shown in Figure 15 and Figure 16, respectively. In Figure 15, about 16.7% of the estimated positions are located on the wrong side of the road before using the proposed RAIM. With the adding of RAIM, it is reduced to only 8.3%. Note that the most of the 8.4% reduction of the point with larger error are corrected to about 4 and 5 m. Note that the RAIM method can improve the proposed 3D map only in the case of receiving the multiple NLOS reflections or the abnormal measurements. As a result, positioning solutions with less than 3 m positioning error both before and after using RAIM are very similar, which account for 53.8% and 58.4% of solutions, respectively. In the case of the deep urban canyon, almost half of the solutions estimated by the 3D map method are on the wrong side of the street, as shown in Figure 16. This can be reduced to 13.7% if the RAIM satellite selection is applied. The positioning solutions within a level of 3 m lateral positioning error both before and after using RAIM are 10.2% and 61.5%, respectively. This result shows the RAIM FDE is essential for the proposed 3D map positioning method, especially in the deep urban canyon environment.

### 5.4. Horizontal Positioning Error Analysis by Static Data

**Figure 17:**
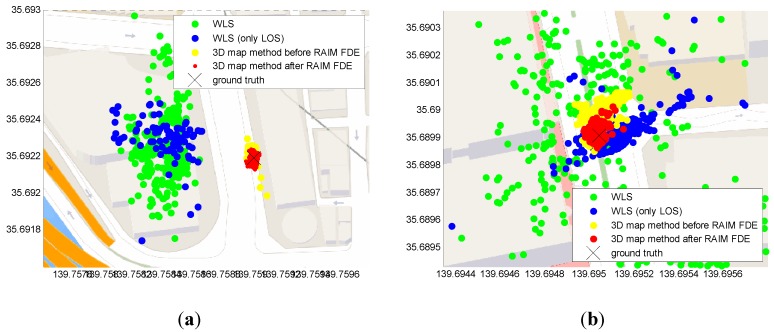
Positioning Results of the static tests using different methods in the (**a**) middle and (**b**) deep urban canyons.

This subsection discusses the horizontal positioning error, which contains both the lateral and longitudinal positioning error of the proposed method. We use static data in this discussion because it is difficult to obtain a perfect ground truth of dynamic pedestrian trajectory. Figure 17 shows the static positioning results in both the middle and deep urban canyon environments. In the case of the middle urban canyon, it is obvious that the positioning results of the conventional WLS using only LOS are located on wrong side of the street. Instead, the proposed 3D map method can estimate the result with correct side of the street. To compare the result of the 3D map method before and after the RAIM satellite selection methods, the red points are denser than the yellow points, not only in lateral but also in longitudinal directions. In Table 5, the RAIM satellite selection method slightly improves the 3D map method in the middle urban scenario; however, it improves the 3D map method greatly in the deep urban canyon case. The means of positioning error are about 11.6 and 3.82 m before and after using the RAIM, respectively. Comparing Table 4 and Table 5, the improvements using the RAIM method are about 4.9 m in the lateral error and 7.8 m in horizontal error. Therefore, we can conclude that the proposed RAIM method can reduce not only lateral but also longitudinal positioning error.

**Table 5:** Horizontal positioning performance of the static tests using the proposed 3D map method before and after using the RAIM method.

Methods	Middle Urban		Deep Urban	
	Mean (m)	Std (m)	Mean (m)	Std (m)
3D map method	3.62	2.44	11.60	4.18
3D map method with RAIM FDE	3.20	1.27	3.82	3.23

## 6. Conclusions

This paper proposed a RAIM satellite selection method for the developed 3D building model based positioning method. This RAIM method is capable of excluding the measurements that are inconsistent with the simulated pseudorange measurements by the 3D building model and ray-tracing technique. These inconsistent pseudorange measurements usually suggest that the received raw pseudorange measurement is a double or multiple reflected NLOS signals or has strong multipath effects, as shown in the experimental results. By excluding the abnormal NLOS signals, the lateral positioning error mean of the proposed 3D map pedestrian positioning method can be reduce from about 3.97 to 2.96 m and 8.78 to 3.85 m in the middle and deep urban canyons, respectively. In addition, the RAIM satellite selection method can also reduce about 8.4% and 36.2% of the positioning solutions with large errors (points that estimated the wrong side of the road) in the middle and deep urban canyon environments, respectively. These results indicate that the proposed RAIM method is more efficient in the deep urban canyon because double reflected NLOS are more frequently observed. Finally, the lateral positioning errors are less than 3 m in around 60% of the solutions of the proposed 3D building model based positioning method in both middle and deep urban canyons.
